# Pharmacodynamics of the Glutamate Receptor Antagonists in the Rat Barrel Cortex

**DOI:** 10.3389/fphar.2018.00698

**Published:** 2018-07-03

**Authors:** Daria Vinokurova, Andrey V. Zakharov, Julia Lebedeva, Gulshat F. Burkhanova, Kseniya A. Chernova, Nailya Lotfullina, Rustem Khazipov, Guzel Valeeva

**Affiliations:** ^1^Laboratory of Neurobiology, Kazan Federal University, Kazan, Russia; ^2^Mediterranean Institute of Neurobiology – National Institute of Health and Medical Research, Aix-Marseille University, UMR1249, Marseille, France

**Keywords:** glutamate receptor antagonists, barrel cortex, epipial application, drug delivery, sensory-evoked potential, fast oscillations, slow wave activity

## Abstract

Epipial application is one of the approaches for drug delivery into the cortex. However, passive diffusion of epipially applied drugs through the cortical depth may be slow, and different drug concentrations may be achieved at different rates across the cortical depth. Here, we explored the pharmacodynamics of the inhibitory effects of epipially applied ionotropic glutamate receptor antagonists CNQX and dAPV on sensory-evoked and spontaneous activity across layers of the cortical barrel column in urethane-anesthetized rats. The inhibitory effects of CNQX and dAPV were observed at concentrations that were an order higher than in slices *in vitro*, and they slowly developed from the cortical surface to depth after epipial application. The level of the inhibitory effects also followed the surface-to-depth gradient, with full inhibition of sensory evoked potentials (SEPs) in the supragranular layers and L4 and only partial inhibition in L5 and L6. During epipial CNQX and dAPV application, spontaneous activity and the late component of multiple unit activity (MUA) during sensory-evoked responses were suppressed faster than the short-latency MUA component. Despite complete suppression of SEPs in L4, sensory-evoked short-latency multiunit responses in L4 persisted, and they were suppressed by further addition of lidocaine suggesting that spikes in thalamocortical axons contribute ∼20% to early multiunit responses. Epipial CNQX and dAPV also completely suppressed sensory-evoked very fast (∼500 Hz) oscillations and spontaneous slow wave activity in L2/3 and L4. However, delta oscillations persisted in L5/6. Thus, CNQX and dAPV exert inhibitory actions on cortical activity during epipial application at much higher concentrations than *in vitro*, and the pharmacodynamics of their inhibitory effects is characterized by the surface-to-depth gradients in the rate of development and the level of inhibition of sensory-evoked and spontaneous cortical activity.

## Introduction

The epipial application of pharmacological agents is widely used for drug delivery into the cortex (Pockberger et al., 1984; Andreasen et al., 1989; Hablitz and Sutor, 1990; Kohling et al., 1993; Conti and Minelli, 1994; Ikeda et al., 2002). This approach appears to be particularly useful for compounds, which are not very permeable through the brain–blood barrier (BBB), and therefore are not effective in systemic delivery. While in a clinical setting the use of epipial drug delivery is limited, this approach may be of interest during drug development as the BBB-impermeable molecule can easily be tested in various disease models with future development of BBB-permeable precursor forms or other delivery methods if the compound shows its efficacy. However, the primary requirement for using epipial application is to know how well the drug penetrates into the brain tissue after application, what the time course of penetration at different depths is, and what drug concentrations should be used during epipial application for effective concentrations at the cortical depth.

During epipial application, drugs passively diffuse through the cortical tissue mainly via extracellular space compartments (Ohata and Marmarou, 1992; Groothuis et al., 2007). However, the diffusion of molecules within the cortical tissue is more complex than diffusion in a free medium. The diffusion within brain tissue is known to be constrained by many factors including tortuosity of extracellular space, direction of axon bundles, uptake into cells, transient binding to receptors or transporters, interaction with negative-charged elements of the extracellular matrix, trapping of molecules in dead-space microdomains, solubility in lipids and electrical charge of diffusing molecules (Sykova and Nicholson, 2008). Therefore, the effective diffusion coefficients for compounds in brain tissue are, on average, half of that in the free medium and may vary depending on brain region and animal age (*ibid*). In keeping with the relatively slow diffusion rates and the thickness of the cortex, drug concentration may develop at different rates and attain different levels at different depths of the cortex. This raises the question of the pharmacodynamics of drugs during epipial application. To elucidate this question, we have chosen a combination of the glutamate ionotropic AMPA/kainate and NMDA receptor antagonists CNQX and dAPV as a model drug, using the spontaneous and sensory-evoked activity in different cortical layers as a readout of their penetration into the cortex.

AMPA/kainate and NMDA receptors are two types of ionotropic glutamate receptors at glutamatergic synapses, pharmacological blockade of which with the selective antagonists CNQX (Honore et al., 1988) and dAPV (Davies et al., 1981) completely suppresses spontaneous and evoked excitatory glutamatergic synaptic transmission (Neuman et al., 1988; Andreasen et al., 1989; Hablitz and Sutor, 1990; Long et al., 1990; Llano et al., 1991; Traynelis et al., 2010). Glutamatergic synapses are critical for generation of the network driven activities involving local and large-scale intracortical excitatory connections and thalamic glutamatergic inputs. In the barrel cortex, which has highly organized excitatory connectivity within and between cortical layers, internally generated spontaneous activity and sensory evoked responses mediated by the thalamus display patterned cross-layer network dynamics (Kandel and Buzsaki, 1997; Steriade, 2001; Buzsaki, 2006; Sakata and Harris, 2009). For example, in the rodent barrel cortex, whisker driven thalamic inputs that mainly project to L4 neurons, are sequentially processed via the canonical L4 – L2/3 – L5 glutamatergic pathway [for review, (Douglas and Martin, 2004; Brecht, 2007; Petersen, 2007; Feldmeyer, 2012; Feldmeyer et al., 2013)]. Also, direct input from the thalamus drives activity in the infragranular layers (Bureau et al., 2006; Brecht, 2007; Meyer et al., 2010; Oberlaender et al., 2012; Constantinople and Bruno, 2013; Crocker-Buque et al., 2015). Activation of the thalamic synaptic inputs and intracortical synapses, as well as voltage-gated conductances in the pre- and postsynaptic neurons underlie transmembrane currents that are at the origin of the extracellular local field potentials (LFPs) recorded at different depths of the cortical column during spontaneous and sensory-evoked activities (Buzsaki et al., 2012).

In the present study, we attempted to determine the effective concentration, exposure times, and inhibition levels produced by epipially applied CNQX/dAPV in different layers of the cortical barrel column. Also, analyzing CNQX/dAPV effects through the time course of their penetration into the cortex we made several observations that could be of interest in cortical barrel network physiology.

## Materials and Methods

### Ethical Approval

This work has been carried out in accordance with EU Directive 2010/63/EU for animal experiments, and all animal-use protocols were approved by the French National Institute of Health and Medical Research (INSERM, protocol N007.08.01) and Kazan Federal University on the use of laboratory animals (ethical approval by the Institutional Animal Care and Use Committee of Kazan State Medical University N9-2013).

### Surgery

Wistar rats of either sex from postnatal day P19–39 (P0 = day of birth) were used. The rat cerebral cortex acquires the diffusion characteristics of the adult brain by P20–P23 (Lehmenkuhler et al., 1993; Vorisek and Sykova, 1997b; Mazel et al., 2002). In addition, we observed no correlation between animal age and the magnitude of the CNQX/dAPV effect, both on L4 sensory evoked potential (SEP) amplitude (*r* = 0.09, *p* = 0.80) and L4 evoked multiple unit activity (MUA) (*r* = -0.47, *p* = 0.14) within a given age range. Therefore, P19–39 animals were pooled into one group. Surgery was performed under isoflurane anesthesia (4% for induction, 2% for maintenance), and urethane (1 g/kg, i.p.) was injected at the end of surgery. The skull of the animal was cleaned of skin and periosteum, dried and covered with cyanacrylamide glue except for a 4–9 mm^2^ window above the left barrel cortex. Then the skull was covered by dental cement (Grip Cement, Dentsply Sirona, Milford, DE, United States). A metal ring (15 mm inner diameter) was fixed to the rat’s head by dental cement. The area inside the ring was left cement-free. After surgery, the animals were warmed, and left for 1 h to recover. The head was attached to a ball-joint holder by the metal ring. During recordings animals were heated via a thermal pad (35–36°C). A chlorided silver wire, placed in the visual cortex, served as the ground electrode.

A cranial window ∼3–4 mm in diameter was drilled above the barrel cortex (window edges: AP -0.5 to -4.5 mm; lateral 2.5–6.5 mm from bregma) (Paxinos and Watson, 2007; Khazipov et al., 2015), and a 1–2 mm-long dura incision was made in the middle of the cranial window using a 27G needle. Dura dissection was done carefully to avoid bleeding and the formation of blood clots on the brain surface, which could obstruct drug diffusion. The cranial window was encircled with a 1 mm-high cement wall to form an epipial chamber. Artificial cerebrospinal fluid (ACSF) during control recordings and CNQX/dAPV dissolved in ACSF were applied to the epipial chamber at 20–40 μl volumes every 5–10 min.

### Extracellular Recordings

Extracellular LFPs and MUA were recorded from a single barrel column using 16-site linear silicon probes (100 μm separation distance between recording sites, NeuroNexus Technologies, Ann Arbor, MI, United States). The probe was inserted vertically to the cortical surface to a depth of 1300–1900 μm (depending on animal age). The signals from extracellular recordings were amplified and filtered (×10,000; 0.15 Hz–10 kHz) using a Digital Lynx (Neuralynx, United States) amplifier, digitized at 32 kHz and saved on a PC for *post hoc* analysis. The whiskers were trimmed to a length of 0.8–1.5 mm and were stimulated using a piezoelectric bending actuator (Noliac, Denmark) using 200 ms square pulses with 5–10 s intervals. A needle (22G) was glued to the end of piezo actuator and the tip of the whisker was inserted into the blunt tip of the needle. The principal whisker (PW) was identified by the shortest latency MUA responses in layer 4 evoked by single whisker deflection.

### Preliminary Selection of the Concentrations and the Mode of Application

To estimate the concentrations of CNQX and dAPV which efficiently inhibit cortical activity, we first applied drugs using the “superfused cortex” preparation (Khazipov and Holmes, 2003; Minlebaev et al., 2007), where the dura is completely removed within the entire cranial window and the cortical surface is continuously superfused. Superfusion of the cortex with 40 μM CNQX/100 μM dAPV, which is more than twice the concentration that completely suppress cortical activity in L4 in neonatal rats (Minlebaev et al., 2007, 2009), caused reduction of the L4 SEP slope to only 60 ± 17% (*n* = 4; *p* = 0.13), and suppression of spontaneous L4 MUA to only 27 ± 7% (*n* = 4; *p* = 0.13) of control values as estimated at 45–60 min after drug application. These moderate effects likely reflect a deceleration of drug diffusion with age (Lehmenkuhler et al., 1993; Vorisek and Sykova, 1997b). A further increase of drug concentrations to 80 μM CNQX/200 μM dAPV also failed to completely inhibit activity in L4 (not shown). As further increase of concentration would be extremely wasteful if applied via perfusion, we next tested two concentrations using static epipial drug application, 170 μM CNQX + 700 μM dAPV and 0.5 mM CNQX + 2 mM dAPV. We found that the reduction of L4 SEP slope to 18 ± 17% (*n* = 4) of control values produced by 170 μM CNQX/700 μM dAPV did not significantly differ (*p* = 0.83) from SEP slope reduction to 2 ± 2% (*n* = 9) produced by 0.5 mM CNQX/2 mM dAPV. Similarly, the inhibition of spontaneous L4 MUA to 7 ± 6% and 3 ± 1% of control values caused by these two combinations of drug concentrations, respectively, were not significantly different (*p* = 0.70). Hereafter we describe the effects of epipially applied CNQX and dAPV at these two concentrations with the results pooled together in group statistics.

### Histology

After recordings the animals were deeply anesthetized with urethane (3 g/kg, intraperitoneally) and perfused through the left cardiac ventricle with 4% paraformaldehyde and 1% glutaraldehyde. The brains were removed and left for fixation in the solution containing glutar- and paraformaldehyde at room temperature. Then the brains were rinsed in PBS and cut into 100 μm-thick slices using a Vibratome (Leica Biosystems, Wetzlar, Germany). The slices, cut in the coronal plane, were used for identification of probe position. Electrode positions were identified from the DiI tracks overlaid on microphotographs of the sections in oblique light or after cresyl-violet staining and depth of the short-latency spikes during sensory-evoked responses.

### Data Analysis and Statistics

Raw data were preprocessed using custom-developed routines in MATLAB environment (MathWorks, United States). The wide-band signal was down-sampled to 1000 Hz and used as the LFP signal. Positive polarity is shown as up in all figures. For action potential detection, the raw wide-band signal was filtered (bandpass 300–5000 Hz) and negative events exceeding five standard deviations calculated over the most silent 1 s length segment of the filtered trace were considered as spikes (MUA). SEPs were detected as the first LFP troughs of sensory evoked responses. LFPs and extracellular spikes were analyzed by custom-written, MATLAB-based programs. Current-source density (CSD) analysis across cortical depth was used to eliminate volume conduction and localize synaptic currents. CSD was computed according to a differential scheme for the second spatial derivative along recording sites. The up-states were detected by deep channels located in L6. Negative LFP deflections exceeding 100 μV and accompanied by MUA were considered as up-states. The early and late components of evoked MUA response were calculated individually for each animal because of slight variations in the temporal properties of sensory response across animals. The early evoked MUA was defined as a time interval where the density of L4 spikes persisting after the full blockade of SEP by CNQX/dAPV exceeded 10% of the level of peak MUA density. Thus, the average onset and duration of the early MUA period were of 7.0 ± 0.2 and 4.3 ± 0.5 ms, respectively (*n* = 11). The time point corresponding to a negative extremum of derivative from evoked MUA density in control was chosen as the onset of the late evoked MUA period, and, on average, accounted for 5.0 ± 0.3 ms after the end of the early evoked MUA period. The late evoked MUA was assessed within the following 20 ms time window.

Statistical analysis was performed using the Matlab Statistics toolbox. The two-side Wilcoxon rank sum test was performed to assess the significance of differences between groups of data with the level of significance kept at *p* < 0.05. Pearson’s correlation coefficient (*r*) was used to describe linear relation between variables.

### Drugs

Urethane, CNQX (6-Cyano-7-nitroquinoxaline-2,3-dione), dAPV (D(-)-2-Amino-5-phosphonopentanoic acid) and lidocaine *N*-ethyl bromide solution were purchased from Sigma. Isoflurane was purchased from Baxter.

## Results

We addressed the pharmacodynamics of the ionotropic glutamate receptor antagonists (170–500 μM CNQX and 0.7–2 mM dAPV) after their epipial administration using linear multisite silicone probe recordings of the LFP signals and MUA at different depths of the cortical barrel column. As a readout of the drug penetration we used several parameters of sensory-evoked responses to brief mechanical stimulation of the PW and spontaneous activity in different cortical layers. These parameters included: (i) slope and amplitude of the SEP; (ii) early and late components of the sensory-evoked MUA response; (iii) characteristics of spontaneous delta-wave activity and frequency of spontaneous MUA. Each of these parameters, as well as the time for attaining half-maximal effect (T_1/2_) and the steady state levels of inhibition were calculated for different cortical layers through the time course of 1 h long drug application.

### SEP

**FIGURE 1:**
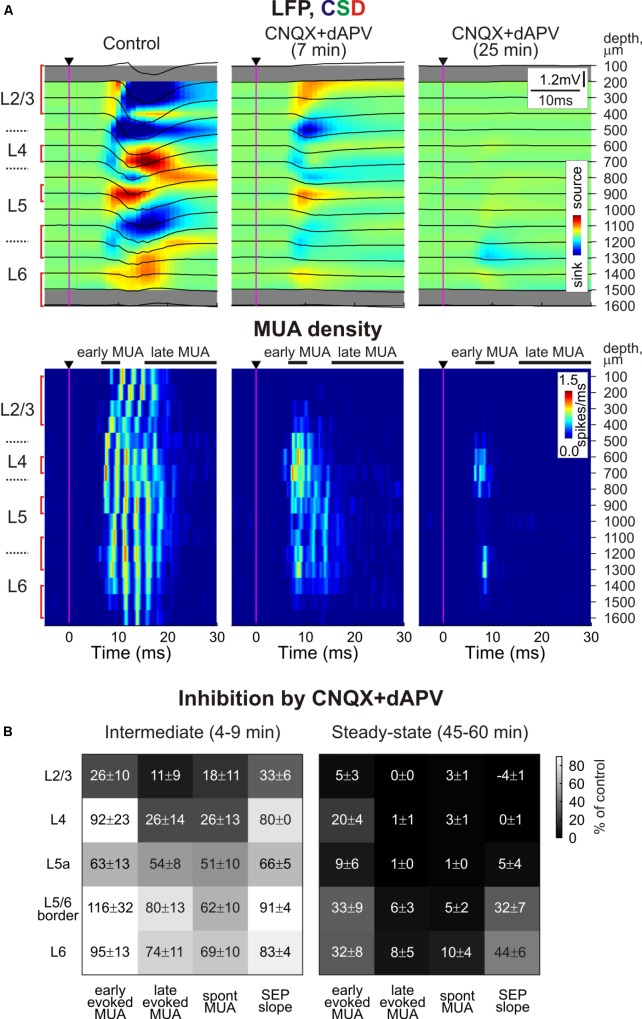
Depth profile of CNQX and dAPV action on barrel cortex activity. **(A)** *Top*, Stimulus-triggered LFP averages (black traces) of sensory responses evoked by principal whisker deflection across cortical depths of corresponding barrel column overlaid on color-coded current source density plot (CSD) in control, 7 and 25 min after 500 μM CNQX and 2 mM dAPV application (25 responses were averaged for each CSD plot). *Bottom*, corresponding stimulus-triggered averages for MUA. Stimulus onset is indicated by the black arrowhead and vertical magenta line. The cortical layer borders are shown left of the CSD and MUA density plots, and recording electrode depths are shown on the right. Red square brackets indicate the electrodes of the recording that were used for building group data plots on panel **(B)**. The gray bars above MUA density plots limit the time windows for early and late MUA density calculation. **(B)** Group data (from 11 animals and two concentrations, 170 μM CNQX/700 μM dAPV and 0.5 mM CNQX/2 mM dAPV) on the magnitude of CNQX and dAPV effect on early and late components of the sensory-evoked MUA, spontaneous MUA and SEP slope at the beginning of drug application (4–9 min, the time point corresponding to SEP slope reduction by 20% of control values) and at the end of 1 h of application (45–60 min). Note the gradual development of CNQX/dAPV effect from superficial toward deep cortical layers, and the persistence of early evoked L4 spikes after full blockade of SEP and nearly complete block of evoked MUA in L2/3 and L5a.

**FIGURE 2:**
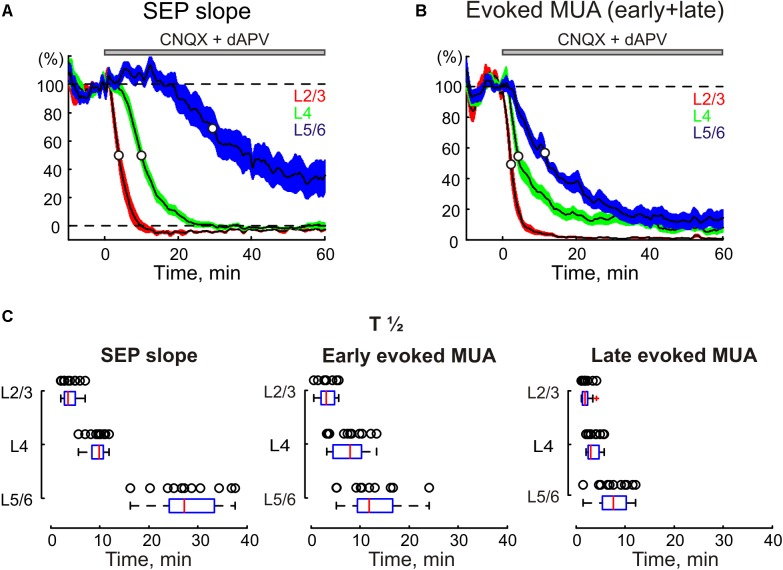
The time course of CNQX and dAPV effect on sensory evoked responses through the depth of the barrel cortex. The decrease in SEP slope **(A)** and MUA during SEP **(B)** in different layers of cortical barrel column following application of CNQX and dAPV. The application time is shown with a gray bar above the plot. White circles on the curves indicate a half-maximal effect time (T½). The colored areas surrounding each curve show SE bands. **(C)** Statistical data on T½ of CNQX and dAPV effect on SEP slope (*left*), early evoked MUA (*middle*) and late evoked MUA (*right*) across cortical layers. The open circles correspond to individual experiments. The medians of boxplots are shown by red lines. **(A–C)**, pooled data from 11 animals and two concentrations, 170 μM CNQX/700 μM dAPV and 0.5 mM CNQX/2 mM dAPV.

**FIGURE 3:**
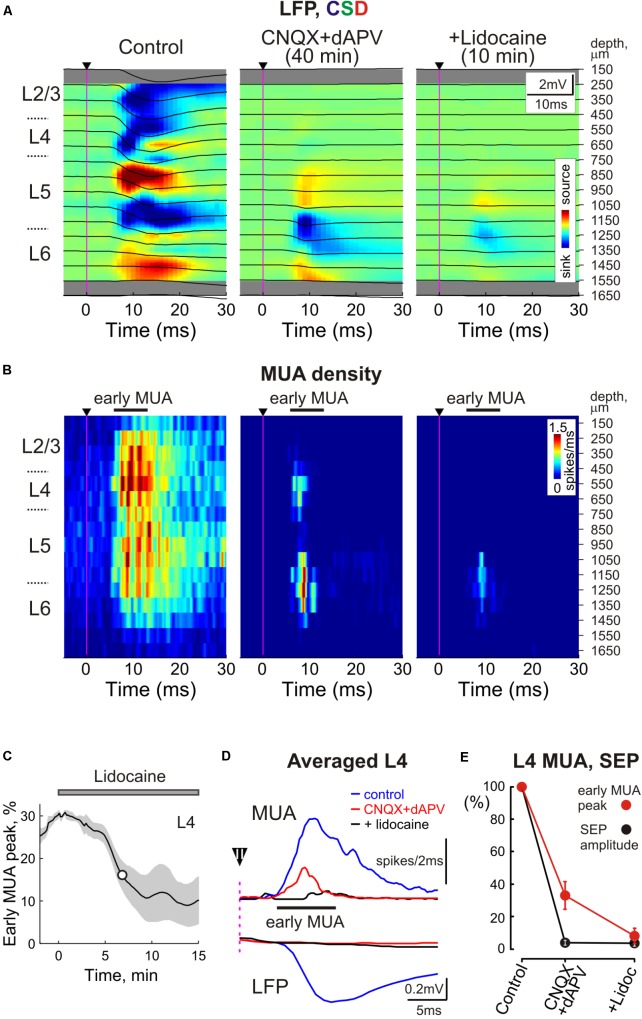
Sensory evoked activity resistant to ionotropic glutamate receptor antagonists in the thalamorecipient cortical layers. **(A)** *Top*, Stimulus-triggered LFP averages (*n* = 30 responses; black traces) of sensory responses evoked by principal whisker deflection across cortical depths of the corresponding barrel column overlaid on color-coded current source density plot (CSD) in control, 40 min after 500 μM CNQX and 2 mM dAPV application, and 10 min after consecutive 2% lidocaine application. **(B)** Corresponding stimulus-triggered averages for MUA (*n* = 30 responses). Stimulus onset on **(A,B)** is indicated by the black arrowhead and vertical magenta line. The cortical layer borders are shown left of the CSD and MUA density plots, and recording electrode depths are shown on the right. **(C)** The time course of early evoked L4 MUA suppression by 2% lidocaine epipially applied (gray bar above the plot) after the full blockade of SEP by CNQX/dAPV. The shaded area around the curve show SE bands (*n* = 4 animals). The white circle on the curve indicates a half-maximal effect time (T½). **(D)** Averaged L4 LFP and MUA in control, in the presence of CNQX and dAPV, and after lidocaine application. Before averaging, individual LFP and MUA curves were aligned by the onset of SEP. Black arrowhead with an error bar above the dashed magenta line shows a mean time with SE between the stimulus and SEP onset. **(E)** Normalized mean values of SEP amplitude and early MUA peak in cortical L4 in control, in the presence of CNQX and dAPV, and after lidocaine application. **(D,E)** Show averaged data from 11 animals and two concentrations, 170 μM CNQX/700 μM dAPV and 0.5 mM CNQX/2 mM dAPV.

Under control conditions, PW deflection evoked SEP with a characteristic CSD profile with two early sinks in L4 and the L5/6 border followed by sinks in L2/3 and L5 (**Figures 1A**, **3A**; Di et al., 1990; Castro-Alamancos and Oldford, 2002; Roy et al., 2011; Reyes-Puerta et al., 2015). The largest sensory response was observed in L4, where average L4 SEP amplitude and slope were of 724 ± 145 μV and 197 ± 41 μV/ms, respectively (*n* = 11). After epipial combined CNQX and dAPV application (170–500 μM CNQX and 0.7–2 mM dAPV), the inhibitory effect of the blockers on SEP was first observed in superficial layers 2/3, and then it successively involved L4 and L5/6 (**Figures 1**, **2**). The T_1/2_ of SEP slope inhibition in L2/3 and L4 were 3.9 ± 0.5 and 9.4 ± 0.5 min after epipial application of blockers, respectively, while in L5/6 it accounted for 28.0 ± 1.9 min (*n* = 11; **Figures 2A,C**). The level of SEP inhibition at 45–60 min of application was stronger at the superficial than deep layers attaining -4 ± 1%, 0 ± 1%, and 32 ± 7% of the control values in L2/3, L4, and L5/6 (L5 and L6 pooled together), respectively (*n* = 11; **Figure 2A**). It is noteworthy that the level of SEP inhibition in the infragranular layers also showed a surface to depth gradient from L5a to 6B (**Figure 1B**, right panel).

### Sensory-Evoked MUA

In parallel with a reduction of SEP, epipial CNQX/dAPV application induced an inhibition of sensory-evoked MUA, which also developed earlier and was in general greater at the superficial layers (**Figure 2B**). Thus, the T_1/2_ of sensory-evoked MUA inhibition was 2.3 ± 0.2, 5.3 ± 0.8, and 13.4 ± 2.2 min in L2/3, L4, and L5/6, respectively (*n* = 11). The steady state level of inhibition of sensory-evoked MUA was also achieved earlier in the superficial layers attaining 5 ± 3, 20 ± 4, and 24 ± 6% of control values in L2/3, L4, and L5/6, respectively. Interestingly, the late component of the sensory-evoked MUA response was inhibited faster and more strongly than the early component (**Figures 1B**, **2C**). Also, the early MUA component was almost completely suppressed in L2/3 (*p* = 0.014) and L5a (*p* = 0.002), but it was less affected in L4 (attaining 20 ± 4% of control values compared to 5 ± 3% in L2/3, *p* = 0.001; and to 9 ± 6% in L5a, *p* = 0.002; **Figures 1A,B**).

Paradoxically, the early MUA component in L4 persisted even after complete suppression of the L4 SEP implying that the spikes at the onset of the sensory-evoked response can occur despite complete blockade of thalamocortical synaptic transmission (**Figures 1**, **3**). In individual experiments, early evoked L4 MUA density in the presence of CNQX/dAPV varied from 19 to 54% of the control values with the average value of 38 ± 3% (average early MUA density was 2.7 ± 0.4 spikes/ms in control conditions and 1.1 ± 0.2 spikes/ms after drug application; *n* = 11; *p* = 0.003; **Figure 3C**). The majority of the CNQX/dAPV insensitive L4 spikes fired mainly within a time window corresponding to the rising phase of evoked MUA in control peaking at 7.8 ± 0.3 ms after the stimulus that was 0.5 ± 0.1 ms earlier than the peak of evoked MUA response in control conditions (*n* = 11; *p* = 0.006; **Figure 3D**). We hypothesized that this CNQX/dAPV-resistant component of the sensory evoked early MUA response could be generated by spikes in thalamocortical axons. In keeping with this hypothesis, after addition of the voltage gated sodium channel blocker lidocaine (2%) to the epipial solution, the peak of the CNQX/dAPV-resistant early evoked L4 MUA response was suppressed from 31 ± 5% of control values to 8 ± 5% during the 15–25 min after lidocaine application with T1/2 of 8 ± 2 min (*n* = 4; *p* = 0.03; **Figures 3B–E**).

### Fast Oscillations

**FIGURE 4:**
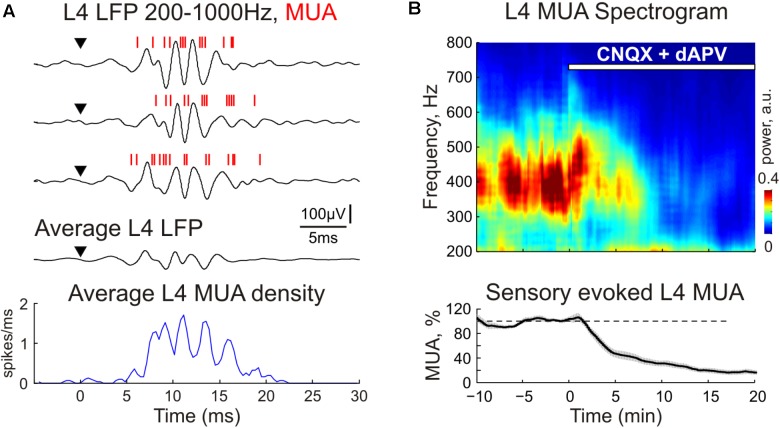
The CNQX and dAPV effect on sensory evoked fast oscillations (FO) in cortical layer 4. **(A)** Three representative traces (after high-pass filtering, 200–1000 Hz) show fast L4 LFP and MUA oscillations evoked by sensory stimulation. Red bars overlaid on the LFP traces are detected spikes (MUA). Stimulus onset is indicated by the arrowhead. Corresponding PSTH of evoked MUA averaged over 30 responses is shown under the LFP traces. **(B)** *Top*, averaged power spectrum of evoked L4 MUA before and during CNQX/dAPV application (application time is shown with a white bar). *Bottom*, the time-course of sensory-evoked (early + late) MUA suppression by CNQX/dAPV. Shaded area shows SE bands. Data were averaged over 11 animals and two concentrations, 170 μM CNQX/700 μM dAPV and 0.5 mM CNQX/2 mM dAPV.

In control conditions, sensory evoked responses were characterized by fast oscillations (FOs) as described earlier (Kandel and Buzsaki, 1997; Jones and Barth, 1999; Jones et al., 2000; Barth, 2003). FOs were observed in all cortical layers, were locked to the stimulus onset and could be clearly seen on the average multiunit response and LFP oscillations after high-pass filtering (**Figures 1A**, **3A**, **4**). A population average L4 MUA power spectrum revealed a peak frequency of FOs at 390 ± 45 Hz (*n* = 11; **Figure 4B**). During CNQX/dAPV application, along with a suppression of SEP and MUA response, L4 FOs reduced in power and duration and were completely eliminated after full blockade of SEP (**Figure 4B**). It is noteworthy, that in individual animals, the power spectrum of L4 multiunit responses displayed two prominent peaks at 451 ± 49 and 645 ± 40 Hz (*n* = 11), both were also suppressed by CNQX/dAPV (data not shown).

### Spontaneous Activity

**FIGURE 5:**
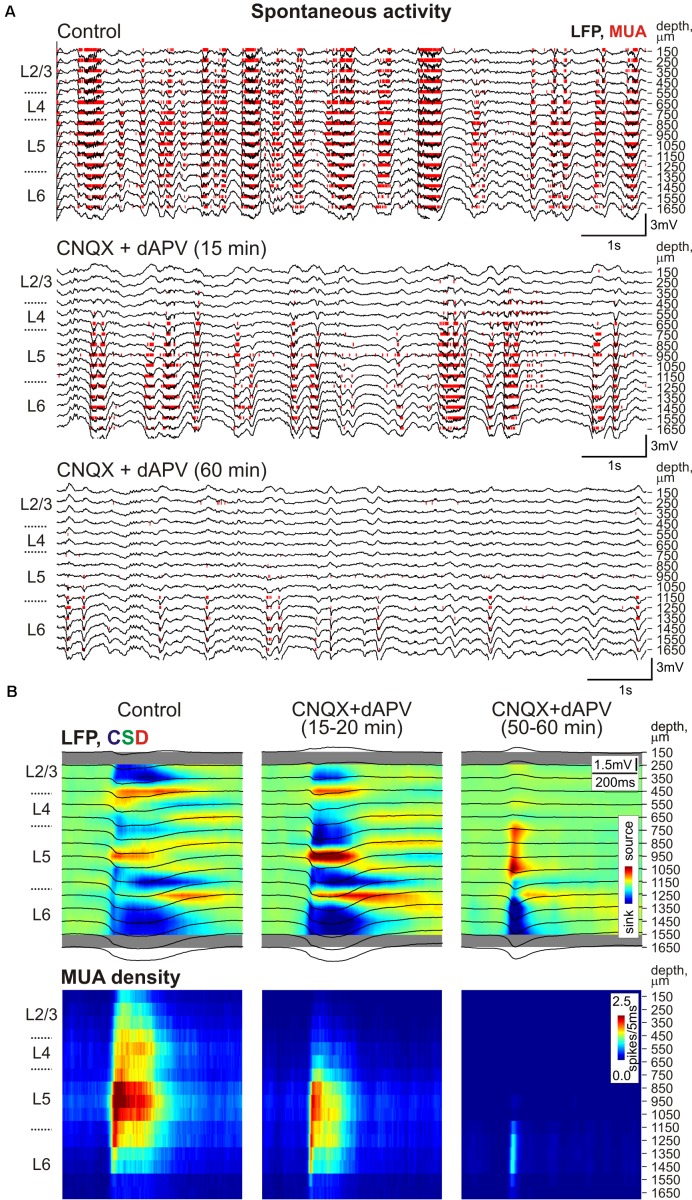
The CNQX and dAPV action on spontaneous activity through the depth of the barrel column. **(A)** Example traces of 16-channel recording of barrel cortex activity in control (*top*), 15 min after epipial application of 500 μM CNQX and 2 mM dAPV (*middle*), and 60 min after drug application (*bottom*). Red bars indicate detected spikes (MUA). **(B)** Corresponding current source density (CSD, *upper panels*) and MUA density (*lower panels*) plots of averaged up-states recorded in control and after drug application (150 events were averaged for each condition). LFP traces (black) are overlaid on color-coded CSD plots. The cortical layer borders on **(A,B)** are shown left of the CSD and MUA density plots, and recording electrode depths are shown on the right.

**FIGURE 6:**
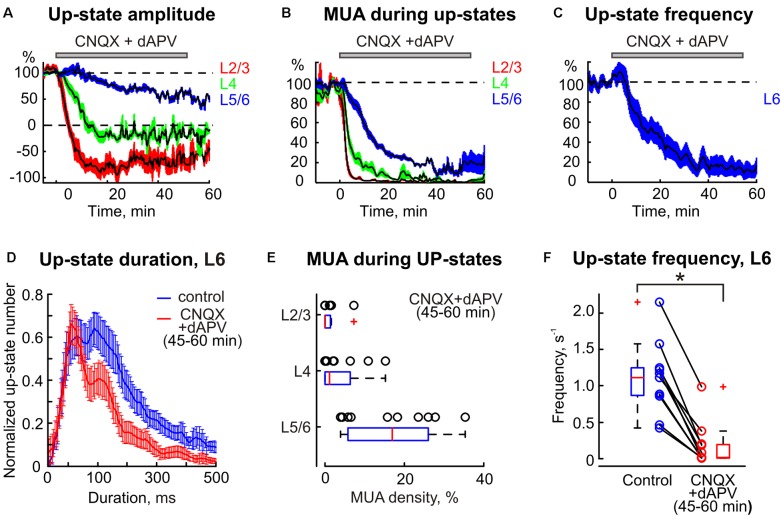
The statistical data on CNQX and dAPV action on spontaneous barrel cortex activity. The decrease in up-states amplitude **(A)**, MUA density during up-states **(B)**, and up-states frequency **(C)** in different layers of the cortical barrel column following application of CNQX/dAPV (application time shown with gray bar above each plot). The colored areas surrounding each curve show SE bands. **(D)** The up-state duration distribution in control and after the drugs application. **(A–D)** Show averaged data from 11 animals and two concentrations, 170 μM CNQX/700 μM dAPV and 0.5 mM CNQX/2 mM dAPV. **(E)** Group data on normalized MUA density in different cortical layers during a steady state of CNQX and dAPV effect. **(F)** Group data on up-states frequency in control and after CNQX/dAPV application. The open circles on **(E,F)** correspond to individual experiments, and the red line on boxplots is a median. ^∗^*p* < 0.05.

Spontaneous activity in the cortical barrel column was characterized by slow wave oscillations. Up-states of slow oscillations typically involved the entire column, and were characterized by sinks and an increase in MUA in all layers with their onset in the infragranular layers (**Figure 5**) as described previously (Sakata and Harris, 2009). Similarly to sensory-evoked responses the spontaneous activity was gradually suppressed by epipial CNQX/dAPV in all layers of the cortical barrel column following a surface-to-depth gradient with faster and stronger inhibition at the superficial layers (**Figures 1B**, **5**, **6**). In L2/3 and L4, where complete SEP inhibition by CNQX/dAPV was observed, LFP deflections during the up-states were first inhibited and then switched polarity from the negative to positive direction, and the up-state related sinks in these layers were converted to sources (**Figures 5**, **6A**). Also, MUA density during up-states decreased in L2/3 from 41.8 ± 10.0 to 0.3 ± 0.2 spikes/s after drug application (*p* = 0.001) and in L4 from 106 ± 17 to 4 ± 2 spikes/s (*p* = 0.001) (*n* = 11; **Figures 6B,E**). During 1 h of CNQX/dAPV application, up-states were reduced yet persisted, maintaining negative LFP polarity, sinks, and MUA activation in the infragranular layers (**Figure 5**). In L6, the up-states frequency decreased from 1.1 ± 0.1 s^-1^ in control conditions to 0.2 ± 0.1 s^-1^ (*n* = 11; *p* = 0.001; **Figures 6C,F**), the up-states amplitude reduced from 376 ± 63 to 236 ± 55 μV, and the up-states duration reduced from 238 ± 19 to 133 ± 12 ms (*n* = 11; *p* = 0.001; **Figures 6A,D**). The MUA frequency during up-states in L5/6 was suppressed by CNQX/dAPV from 106 ± 13 to 15 ± 3 spikes/s (*n* = 11; *p* = 0.001; **Figures 6B,E**). These findings are consistent with the importance of intracortical excitatory connections in the generation of slow wave activity and the initiation of up-states in the infragranular layers (Silva et al., 1991; Sanchez-Vives and McCormick, 2000; Timofeev et al., 2000; Sakata and Harris, 2009).

## Discussion

In the present study, we assessed the time course and the magnitude of inhibitory effects produced by epipially applied ionotropic glutamate receptor antagonists on sensory evoked and spontaneous network activity through the depth of the rat barrel cortex *in vivo*. The described pharmacodynamics of these drugs may also allow the prediction of diffusion dynamics of other drugs with similar diffusion coefficients.

### Development of the Inhibitory Effects of CNQX/dAPV Through the Cortical Depth

In our experiments, the concentration of CNQX/dAPV (170–500 μM CNQX and 0.7–2 mM dAPV) that fully blocked SEP in L2/3 and L4 was more than 10 times the glutamate receptor antagonist concentration generally used for bath application to suppress thalamocortical excitatory synaptic responses in slices *in vitro* (10 μM CNQX and 50 μM dAPV) (Feldman et al., 1998; Laurent et al., 2002), in intact preparations of superfused hippocampus of P15-25 rats *in vivo* (50 μM CNQX) (Khazipov and Holmes, 2003) and in superfusion of the neonatal rat barrel cortex *in vivo* (20 μM CNQX and 80 μM dAPV) (Minlebaev et al., 2007, 2009). The time course of inhibition produced by epipially applied glutamate receptor antagonists was comparable with bath application *in vitro* only in superficial L2/3, in deeper layers the inhibitory effects developed much slower following a surface-to-depth gradient. This likely reflects passive drug diffusion across the cortical depth counterbalanced by their removal from the extracellular space through blood microcirculation. Both of these factors are age-dependent with larger extracellular space volume (Bondareff and Pysh, 1968; Bondareff and Narotzky, 1972; Lehmenkuhler et al., 1993; Sykova et al., 1998, Sykova et al., 2002; Kilb et al., 2006) and less developed vascularization (Keep and Jones, 1990; Yu et al., 1994; Risser et al., 2009) in the immature animals. Together with a thinner cortex in the immature animals, this likely explains the much higher effective antagonist concentrations of the antagonists during their epipial application in older animals compared to neonates.

**FIGURE 7:**
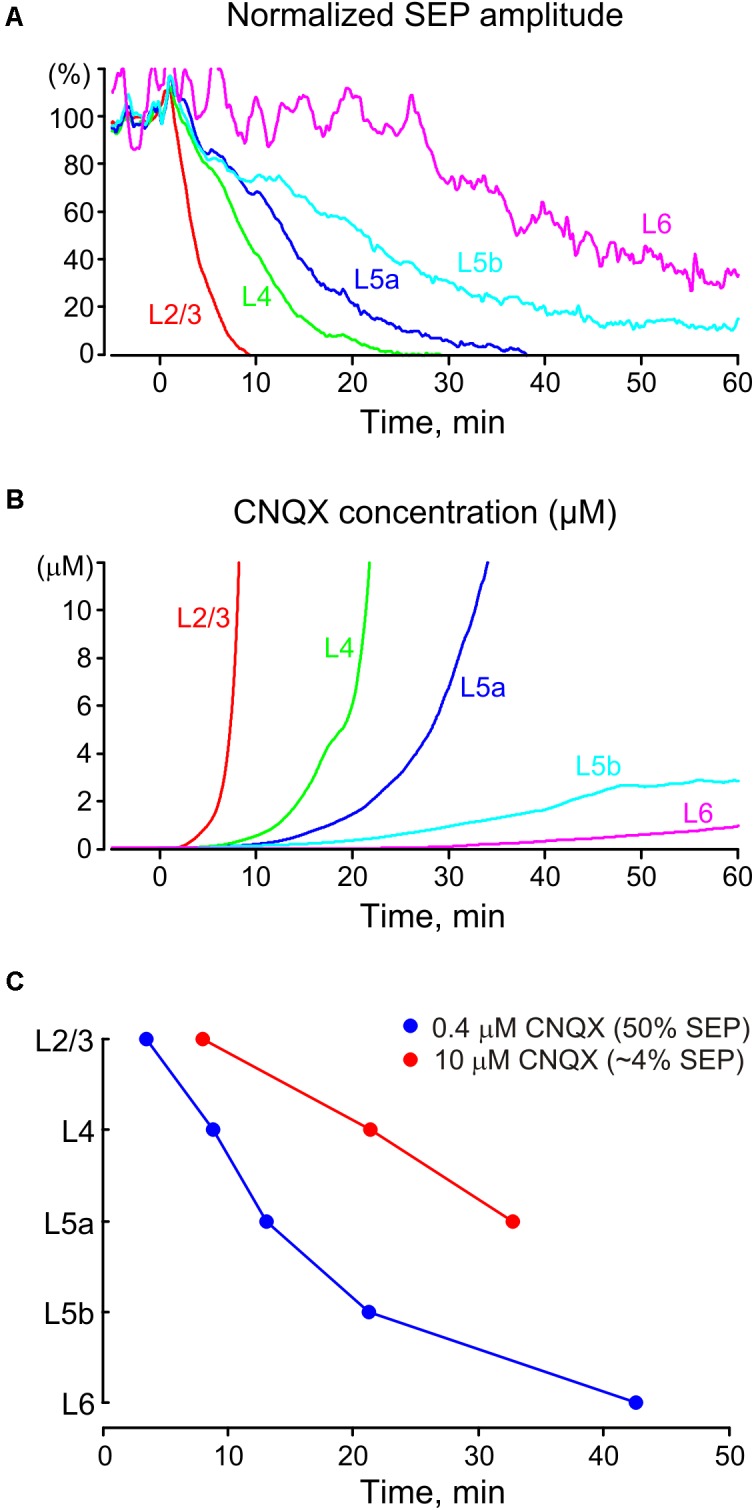
Pharmacokinetics of CNQX and dAPV after epipial application. **(A)** Development of the inhibitory effect of CNQX and dAPV on SEP amplitude across cortical layers over 1 h of application (averaged data from 11 animals). **(B)** Estimation of CNQX concentration dynamics in different cortical layers after epipial drug application. Instant values of CNQX concentration were calculated based on the concentration dependence of CNQX effect on the fast EP SCs with a *K*d of 0.4 μM and Hill coefficient of 1 (Honore et al., 1988; Llano et al., 1991). **(C)** The time points where epipially applied CNQX (170–500 μM) attains *K*d values of 0.4 and 10 μM at different depths of the cortical column.

Various parameters of cortical activity displayed different sensitivity to the glutamate receptor antagonists. At a given cortical depth, spontaneous activity and the late component of sensory-evoked responses were inhibited earlier and stronger than the early component of sensory-evoked responses including SEP slope and amplitude, and early evoked MUA response. This difference likely involves the monosynaptic nature of SEP, an initial part of which is generated by the activation of thalamocortical synapses whereas the late part of the sensory-evoked response and spontaneous activity are polysynaptic events supported by the intracortical connections and therefore are more sensitive to the attenuation of excitatory synapses (Johnston and Brown, 1981). Interestingly, the early evoked MUA response in L4 and the L5/6 border was the most resistant to the glutamate receptor antagonists which is likely due to a contribution of the action potentials in the thalamocortical axon terminals to MUA in the thalamorecipient layers (see below). Also, we observed that sensory-evoked responses persisted in the infragranular layers despite complete suppression of sensory responses in the supragranular layers (e.g., see **Figure 7A**), which is consistent with the direct thalamic input to the infragranular layers (Bureau et al., 2006; Brecht, 2007; Meyer et al., 2010; Oberlaender et al., 2012; Constantinople and Bruno, 2013; Crocker-Buque et al., 2015).

### Pharmacokinetics of the Glutamate Receptor Antagonists After Epipial Application

We further attempted to determine the cross-layer pharmacokinetics of glutamate receptor antagonists after epipial application. With this aim, we assessed SEP amplitude as the most robust parameter of glutamatergic synaptic transmission (**Figure 7A**). The level of SEP inhibition in different layers after epipial application of the glutamate receptor antagonists was further used for an estimation of the instant antagonist concentration in this layer at a given time point. To perform this effect-to-concentration conversion we based our calculations on the concentration dependence of the inhibitory effects of CNQX on evoked fast EPSCs with a *K*d of 0.4 μM and Hill coefficient of 1 (Honore et al., 1988; Llano et al., 1991) according to the formula:

$$A={Kd}^{h}/(c^{h}+{Kd}^{h}),$$

where A is a normalized value of the determined parameter (SEP amplitude), h – Hill coefficient and c – drug concentration.

The results of conversion of the inhibitory effects of CNQX on SEP amplitude to the actual concentration of the drug at different cortical layers are shown on **Figure 7B**. According to this analysis, CNQX concentration progressively increased most rapidly at the superficial layers attaining saturation in L2/3 and L4. The CNQX concentration likely further continued increasing in these layers during the drug application but these values above saturating effects are hard to estimate in our assay. In deep layers, CNQX concentration increased much slower and attained only sub-saturating levels of a few μM that is two orders lower than the drug concentration in the epipial solution. We also estimated the time points after the onset of antagonist application at which CNQX concentration attained the *K*d value of 0.4 and 10 μM corresponding to the inhibition of the SEP amplitude to 50 and 3.8% of the control values, respectively (**Figure 7C**). These estimations also indicated that the effective concentration levels of the antagonist were slowly achieved through the cortical depth during epipial drug application, and the 10 μM concentration level was attained during 1 h of application at only about one half of the cortical depth. Given the remarkably similar diffusion parameters observed in different cortical areas (Vorisek et al., 2002; Slais et al., 2008) of the adult brain, as well as between different brain structures (Nicholson and Phillips, 1981; Svoboda and Sykova, 1991; Lehmenkuhler et al., 1993; Sykova et al., 1994, 1998; Simonova et al., 1996; Vorisek and Sykova, 1997b; Mazel et al., 1998, 2002; Reum et al., 2002) and species (Vargova et al., 2003; Sykova et al., 2005; Homola et al., 2006) the diffusion dynamics of glutamate antagonists and other molecules with similar diffusion properties through the rat somatosensory cortex described in the present study are likely to be relevant for the majority of brain regions. Our results could be of interest for the estimations of pharmacodynamics in other methods of drug delivery including injection into the brain tissue or intraventricular application. It is worthy to note, that drug diffusion dynamics may differ between horizontal and vertical directions in the brain regions exhibiting anisotropic tissue properties (Vorisek and Sykova, 1997a; Mazel et al., 1998; Sykova et al., 1998, Sykova et al., 2002; Vorisek et al., 2002).

### Contribution of Thalamic Axonal Spikes to Cortical MUA

Multiple unit activity is assumed to represent action potentials from multiple neurons whose soma are located near the extracellular recording electrode (Henze et al., 2000; Bartho et al., 2004; Buzsaki et al., 2012). Here, we provide evidence that the action potentials, not only in cortical neurons, but also in thalamocortical axons contribute to MUA in cortical L4. Indeed, we found that spikes with the shortest latency during sensory-evoked responses persist after full blockade of glutamatergic transmission in L4 as evidenced by complete suppression of L4 SEPs. This is in keeping with the findings obtained in the primary somatosensory cortex of piglets using high-resolution magnetoencephalography, where sensory evoked responses consisted of the initial component which was localized in cortical L4 and which was insensitive to glutamate receptor blockade by kynurenic acid, and the subsequent kynurenate – sensitive component (Ikeda et al., 2002). The glutamate receptor antagonist insensitive MUA component which persisted in the sensory-evoked response in L4 has also been reported in the barrel cortex of neonatal rat pups (Minlebaev et al., 2007). This glutamate receptor antagonist resistant MUA was suppressed by voltage-gated sodium channel blockers, lidocaine in the present study and tetrodotoxin in a previous study in rat pups (Minlebaev et al., 2007). Several observations further suggest that this glutamate receptor antagonists resistant MUA represents spikes in thalamocortical axon terminals. Firstly, the mean latency between the stimulus and the peak of early evoked MUA in the presence of CNQX/dAPV was 7.9 ± 0.4 ms, while the latency of spikes recorded in whole-cell configuration from L4 neurons in response to whisker deflection is more than 10 ms (Constantinople and Bruno, 2013). Also, blockade of glutamatergic ionotropic receptors completely suppressed the sensory-evoked postsynaptic response and action potentials in L4 neurons during whole-cell recordings whereas the extracellular sensory-evoked MUA response persisted in the neonatal rat barrel cortex (Minlebaev et al., 2007). Secondly, in the present study, the short latency spikes were mainly restricted to the L4 and L5/6 border where the thalamocortical axon terminals extensively ramify and one could expect efficient summation of the transmembrane currents during action potentials in these compactly organized multiple axonal terminals and generation of large LFP signals (Chmielowska et al., 1989; Lu and Lin, 1993; Bruno and Sakmann, 2006; Bureau et al., 2006; Meyer et al., 2010). Finally, LFP signals generated in L4 by the activity of single thalamocortical neurons are characterized by the short-latency spikes representing afferent volleys followed by glutamatergic postsynaptic current, the latter being selectively suppressed by the intracortical infusion of the AMPA/kainate receptor blocker (Swadlow and Gusev, 2002; Swadlow et al., 2002; Hagen et al., 2017). Taken together, these findings indicate that spikes in thalamocortical axons significantly (∼20%) contribute to the initial part of the sensory-evoked MUA response during extracellular intracortical recordings in L4 (but also likely at the L5/6 border) and this contribution should be considered during MUA analysis. Our findings also suggest that thalamic activation evoked by rapid whisker deflection lasts for only ∼5 ms and the rest of the MUA response is mainly generated by cortical neurons. This is different from the neonatal rat pups, where sensory-evoked MUA response after suppression of the glutamatergic ionotropic receptors lasts for several hundreds of milliseconds in keeping with the long-lasting activity bursts that are evoked in the thalamus by brief sensory stimuli (Minlebaev et al., 2011; Yang et al., 2013). Short duration of the CNQX/APV resistant, presumably thalamocortical axonal activity at the onset of sensory-evoked response in adult animals also suggests that thalamic input only generates the initial part of SEP whereas the late part of the response is supported by the local circuits, and that it only triggers but does not pace FOs that is in keeping with the pivotal roles of the intracortical connections, notably GABAergic synapses by the fast spiking interneurons, in the genesis of FOs (Jones et al., 2000).

## Data Availability

Original and processed data, and signal processing and analysis routines are available on request from the authors.

## Author Contributions

RK conceived the project. DV, JL, NL, GB, and KC performed the experiments. AZ and GV analyzed the data. GV and RK wrote the paper.

## Conflict of Interest Statement

The authors declare that the research was conducted in the absence of any commercial or financial relationships that could be construed as a potential conflict of interest.
